# An Audit of Eye-Protection Practices in ENT-Related Facial Nerve Palsy: A Retrospective Review of Discharge Summaries

**DOI:** 10.7759/cureus.99797

**Published:** 2025-12-21

**Authors:** Saleh Khurshied, Muhammad A Zahid, Hafiz Mahboob Ul Hassan, Hafiza Tehseen Fatima, Asma Afsar, Mehrun Nisa, Hira G Shah

**Affiliations:** 1 Otolaryngology - Head and Neck Surgery, Pakistan Institute of Medical Sciences, Islamabad, PAK; 2 Ophthalmology, Monash Health, Clayton, AUS; 3 Medicine and Surgery, Basic Health Unit Karore, Murree, PAK; 4 Medicine and Surgery, Pakistan Institute of Medical Sciences, Islamabad, PAK; 5 Ophthalmology, Alshifa Trust Eye Hospital, Rawalpindi, PAK

**Keywords:** discharge checklist, facial nerve palsy, prevention of complication, record review, eye care

## Abstract

Background and objective

Facial nerve palsy (FNP) secondary to ENT-related conditions can lead to lagophthalmos and exposure keratopathy, putting patients at risk of vision-threatening complications. Proper documentation of eye-protection measures in discharge summaries is critical for continuity of care and patient safety. This study aimed to evaluate the quality and completeness of eye-care instructions in discharge summaries of ENT-related FNP patients.

Materials and methods

A retrospective review was conducted on 104 discharge summaries from the Department of ENT - Head and Neck Surgery, Pakistan Institute of Medical Sciences, Islamabad, over a 32-month period (from January 2023 to August 2025). Patients included had FNP due to ENT-related causes, while non-ENT etiologies and incomplete records were excluded. A checklist based on ENT UK, National Institute for Health and Care Excellence (NICE), and Royal College of Ophthalmology guidelines was used to assess documentation of eye-protection measures. Discharge summaries were scored from 0 to 6 and classified as poor (0-1), fair (2-3), good (4-5), or excellent (6). Statistical analysis was performed using SPSS v27 (IBM Corp., Armonk, NY).

Results

The mean patient age was 46 ± 13.5 years; 67 (64.4%) were male. The most common etiologies were chronic suppurative otitis media with cholesteatoma (40.5%), head and neck cancer (22.1%), and trauma (16.3%). Documented eye-care measures included daytime lubricating drops (96.2%), eyelid taping (84.6%), follow-up instructions (74.0%), night-time ointment (66.3%), ophthalmology referral (38.5%), and corneal status/lagophthalmos (24.0%). Overall discharge summary quality was excellent in 8.7%, good in 30.8%, fair in 58.7%, and poor in 1.9%. Collapsing the ratings into adequate and inadequate showed that 41 summaries (39.4%) were adequate, and 63 (60.6%) were inadequate.

Conclusion

While basic eye-care instructions were frequently documented, critical elements such as corneal assessment and ophthalmology referral were often missing. Several discharge summaries were inadequate, emphasizing the need for standardized, comprehensive documentation to improve patient safety and reduce preventable ocular morbidity in ENT-related FNP.

## Introduction

Facial nerve palsy (FNP) is a common and significant condition encountered in Ear, Nose, and Throat (ENT) practice, arising from surgical trauma, infections, neoplasms, or idiopathic causes, and often resulting in both functional and cosmetic morbidity [1]. Paralysis of the orbicularis oculi muscle leads to lagophthalmos, which exposes the corneal surface to desiccation and increases the risk of exposure keratopathy, ulceration, and potentially permanent vision loss [2]. Early intervention is therefore essential to preserve visual function.

Initial management generally involves conservative protective strategies, including frequent application of artificial tears, night-time lubricating ointment, eyelid taping, and the use of moisture chambers to maintain corneal hydration and reduce exposure [2]. Temporary external eyelid weights can be effective in acute or reversible cases where dynamic recovery is anticipated [3]. In chronic or severe cases, surgical interventions such as gold or platinum upper-eyelid loading, tarsorrhaphy, or medial canthoplasty may be necessary to restore functional eyelid closure and prevent ongoing corneal damage [4].

Ocular involvement in FNP not only leads to physical complications but also significantly affects patient comfort, daily functioning, and overall quality of life. Recent studies have highlighted that while lubricants are generally well tolerated, interventions such as eyelid taping may reduce adherence, emphasizing the need for individualized management plans [5]. Symptoms, including ocular discomfort, photophobia, and fluctuating vision, can profoundly impact psychological well-being and daily activities in patients with unresolved FNP [4]. Multidisciplinary management involving ENT, ophthalmology, and facial nerve specialists has been shown to improve both functional and visual outcomes [6].

Patients with FNP are particularly susceptible to exposure keratopathy due to incomplete lid closure, paralytic ectropion, and impaired tear film distribution, which may be compounded by meibomian gland dysfunction [7,8]. Despite the existence of clear evidence-based guidelines for eye protection in FNP, documentation of eye-care instructions in discharge summaries remains inconsistent across clinical settings. Discharge certificates are critical for ensuring continuity of care and medico-legal accountability, yet the quality of eye-protection documentation in ENT-related facial palsy has not been well studied.

This study, therefore, aims to evaluate the quality and completeness of eye-protection advice in discharge certificates of patients with ENT-related FNP, using a structured scoring system based on established clinical guidelines.

## Materials and methods

This study was designed as a retrospective review carried out in the Department of ENT - Head and Neck Surgery at the Pakistan Institute of Medical Sciences (PIMS), Islamabad. The review covered a 32-month period from January 2023 to August 2025. During this time, all discharge certificates issued for patients admitted with FNP of ENT-related origin were examined. Patients were included if they had a confirmed diagnosis of facial nerve dysfunction due to an ENT-related cause and were hospitalized under ENT care. These conditions consisted of head and neck tumors requiring management, ear surgeries for acute or chronic ear disease, temporal bone injuries, parotid gland surgery, cholesteatoma, chronic suppurative otitis media (CSOM), acute suppurative otitis media (ASOM), and facial nerve injuries resulting from medical or surgical procedures. Individuals were excluded if their FNP resulted from non-ENT causes such as idiopathic palsy, stroke, diabetes-related neuropathy, or Ramsay Hunt syndrome. Patients not admitted under the ENT service and those with discharge summaries lacking essential information were also excluded.

To assess the thoroughness of eye-care recommendations provided to patients with facial palsy, a detailed evaluation checklist was developed by the authors. The checklist was based on recognized guidelines from ENT UK, the National Institute for Health and Care Excellence (NICE), and the Royal College of Ophthalmology [9-12]. These outline the best practices for preventing corneal complications in patients with incomplete eyelid closure. Checklist items covered documentation of eye-protection instructions, the prescription of lubricating eye drops or ointments, advice regarding eyelid taping or eye patch use, identification of symptoms that require urgent review, recommendations for ophthalmology referral when necessary, and instructions for follow-up care.

A scoring system was created by the authors to rate each discharge certificate according to the checklist. Documents receiving scores of 0 or 1 were regarded as poor, indicating inadequate or missing guidance and increased risk for eye injury. Scores of 2 or 3 were considered fair, representing partial but insufficient information. Scores of 4 or 5 were categorized as good, showing that most recommended elements had been documented, while a score of 6 was designated excellent, reflecting complete adherence to recommended eye-care documentation. For analytical purposes, certificates rated as good or excellent were grouped as adequate, while those rated poor or fair were classified as inadequate.

All extracted data were recorded using a structured form and analyzed with IBM SPSS Statistics for Windows, version 27.0 (IBM Corp., Armonk, NY, 2018). Descriptive statistics, including means, frequencies, and percentages, were used to summarize the findings. Tables and graphical displays were generated to illustrate the distribution of scores and patient characteristics.

## Results

**Figure 1:**
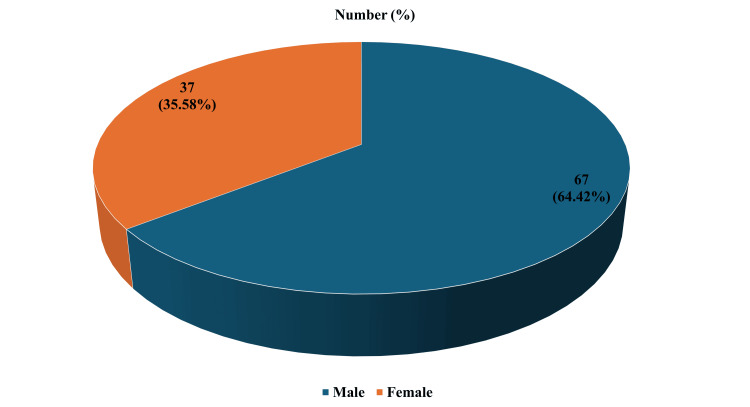
Distribution of gender of the patients included in the study Number of ENT-related FNP-admitted male and female patients over the 32-month study period (January 2023–August 2025), with each gender’s proportion of the total shown in brackets. ENT: Ear, Nose, and Throat; FNP: Facial nerve palsy.

Over a period of 32 months, a total of 104 discharge certificates were reviewed. The mean age ± standard deviation of the patients was 46 ± 13.5 years, with 67 males (64.42%) and 37 females (35.58%), as shown in Figure 1.

The most common causes of ENT-related FNP were CSOM with cholesteatoma (42 cases, 40.48%), head and neck cancer (23 cases, 22.11%), trauma and fractures (17 cases, 16.35%), acute otitis media (9 cases, 8.65%), CSOM without cholesteatoma (7 cases, 6.73%), iatrogenic causes (4 cases, 3.85%), and other minor causes (2 cases, 1.92%). This has been detailed in Table 1.

**Table 1:** Distribution of patients across ENT-related causes of FNP Total number of cases (N): 104 (100%). ENT: Ear, Nose, and Throat; FNP: Facial nerve palsy.

Diagnosis	Number of patients	Percentage (%)
Acute bacterial rhinosinusitis	7	7.14
Allergic fungal rhinosinusitis	5	5.10
Acute invasive fungal rhinosinusitis	9	9.18
Chronic rhinosinusitis	12	12.24
Squamous cell carcinoma maxillary sinus	7	7.14
Adenocarcinoma ethmoid sinus	4	4.08
Other sinonasal malignancies	2	2.04
Orbital cellulitis	11	11.22
Chronic otitis media with complications	10	10.20
Temporal bone malignancy	2	2.04
Parotid malignancies	7	7.14
Thyroid disease	2	2.04
Rhabdomyosarcoma sinus and orbit	1	1.02
Parapharyngeal space mass	3	3.06
Trauma	8	8.16
Acute otitis media/mastoiditis	8	8.16

**Figure 2:**
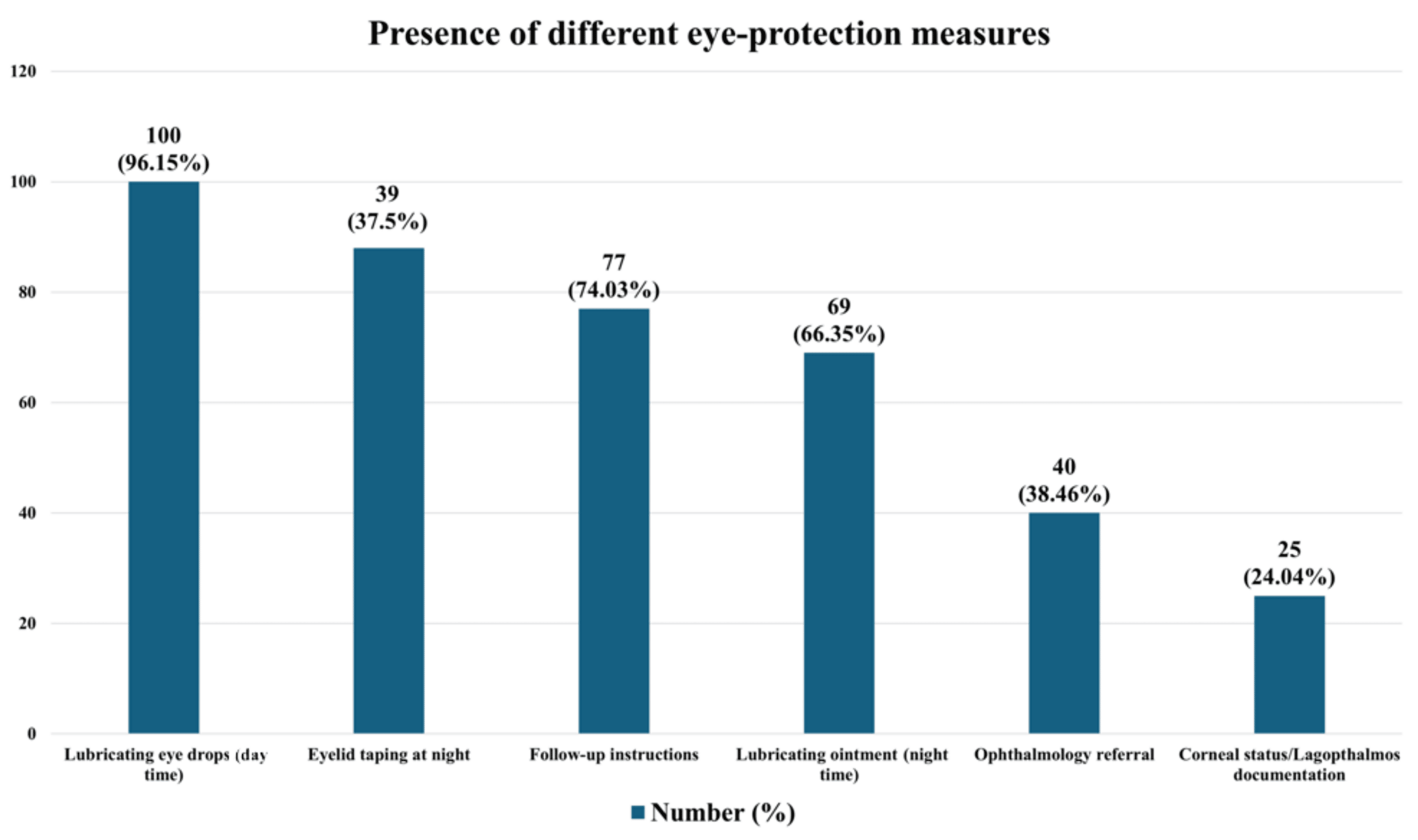
Distribution of documented eye-care measures y-axis (number of cases): The number of certificates with specific eye-care measures documented. x-axis (eye-care measures): The different eye-care measures according to the checklist based on recognized guidelines from ENT UK, the National Institute for Health and Care Excellence (NICE), and the Royal College of Ophthalmology [9-12].

Regarding eye-care measures documented in the discharge certificates, 100 patients (96.15%) were prescribed daytime lubricating eye drops, 69 (66.34%) received night-time lubricating ointment, 88 (84.61%) had eyelid taping recommended at night, 25 (24.04%) had corneal status or lagophthalmos documented, 77 (74.04%) were given follow-up instructions for eye care, and 40 (38.46%) were referred for ophthalmologic assessment as presented in Figure 2.

When evaluating the overall quality of the discharge summaries, only 9 (8.65%) were rated as excellent (score: 6), 32 (30.78%) as good (score: 4-5), 61 (58.65%) as fair (score: 2-3), and 2 (1.93%) as poor (score: 0-1). When the quality ratings were collapsed into adequate (good or excellent) and inadequate (fair or poor), 41 (39.4%) met the acceptable standard, whereas 63 (60.6%) fell below the required level, as presented in Table 2.

**Table 2:** Distribution of quality scores Total number of cases (N): 104 (100%). Self-developed score based on recognized guidelines from ENT UK, the National Institute for Health and Care Excellence (NICE), and the Royal College of Ophthalmology [9-12].

Quality score distribution	Number (%)	Interpretation
Excellent (6)	9 (8.6)	Adequate 41 (39.4%)
Good (4-5)	32 (30.77)	
Fair (2-3)	61 (58.65)	Inadequate 63 (60.6%)
Poor (0-1)	2 (1.92)	

## Discussion

This study evaluated the quality and completeness of documented eye-protection advice in discharge certificates of patients with ENT-related FNP. Our findings indicate that, although many patients received some form of ocular-protection instruction, documentation was inconsistent and often incomplete. This highlights a gap between established clinical guidelines and routine discharge practices.

A large cohort study by Singh et al. [1] reported nearly 50% ocular surface exposure and 15% severe visual impairment in FNP patients, emphasizing that inadequate eye-care instructions may contribute to preventable visual morbidity. While neoplastic etiology was the most common cause of FNP in their study, it was the second most frequent in our cohort. Male predominance was observed in both studies. Portelinha et al. [13] demonstrated that corneal changes can develop rapidly, even within days, underscoring the importance of early intervention for optimal outcomes.

In a study from Saudi Arabia, Al Jaber et al. [14] reported that 70% of physicians were aware of the correct management of FNP, whereas in our study, only about half of the discharge summaries were rated as good to excellent. Liu et al. [15] reported that 64.2% of patients received adequate eye care, compared to approximately 40% in our cohort. Previous research has shown that healthcare practitioners’ knowledge of FNP diagnosis and management significantly influences rehabilitation outcomes [16,17]. Albishi et al. [18] found that 1.3% of participants had low knowledge levels, 56.7% had moderate knowledge levels, and 42% had high knowledge levels, which align with our findings regarding poor, fair, and combined good/excellent documentation.

Regarding etiology, Venugopa et al. [19] identified cholesteatoma as the most common cause of ENT-related FNP, consistent with our findings, while other studies have reported trauma as the predominant cause [20].

This study is among the few to specifically examine the quality of eye-protection instructions in ENT discharge documentation, a critical but often overlooked aspect of patient safety. Our findings highlight several areas for improvement, including the adoption of standardized discharge templates, mandatory inclusion of basic eye-protection advice, clearly defined ophthalmology referral pathways, and targeted education of ENT clinicians on evidence-based eye-care guidelines. Given the high risk of vision-threatening complications reported in the literature [1], improving discharge documentation could directly reduce preventable morbidity.

As a retrospective analysis, the study is limited by its reliance on the completeness and accuracy of existing records. Clinical advice may have been provided verbally but not documented. Additionally, the study did not assess patient adherence to instructions or clinical outcomes after discharge, and it was conducted at a single center, which may limit generalizability.

Based on these findings, we recommend the inclusion of a mandatory eye-protection section in discharge templates, training of junior staff in the management of FNP, and implementation of a standardized eye-care proforma to improve documentation and patient safety.

## Conclusions

The most common cause of ENT-related FNP in this study was CSOM with cholesteatoma. Documentation of corneal status and appropriate ophthalmology referral was missing in the majority of discharge certificates. More than half of the certificates were inadequately written with respect to eye-protection advice, showing that most discharge certificates were deficient with respect to essential advice. These findings underscore that, while basic eye-care recommendations are often recorded, comprehensive evaluation and referral for ophthalmology are frequently omitted, highlighting the need for standardized and thorough discharge documentation for patients with FNP.
